# Identification and Characterization of the Major Porin of Desulfovibrio vulgaris Hildenborough

**DOI:** 10.1128/JB.00286-17

**Published:** 2017-10-31

**Authors:** Lucy Zeng, Etsuko Wooton, David A. Stahl, Peter J. Walian

**Affiliations:** a Molecular Biophysics and Integrated Bioimaging Division, Lawrence Berkeley National Laboratory, Berkeley, California, USA; b Department of Molecular and Cell Biology, University of California, Berkeley, Berkeley, California, USA; c Department of Civil and Environmental Engineering, University of Washington, Seattle, Washington, USA; Michigan State University

**Keywords:** membrane channel proteins, outer membrane proteins, porins

## Abstract

Due in large part to their ability to facilitate the diffusion of a diverse range of solutes across the outer membrane (OM) of Gram-negative bacteria, the porins represent one of the most prominent and important bacterial membrane protein superfamilies. Notably, for the Gram-negative bacterium Desulfovibrio vulgaris Hildenborough, a model organism for studies of sulfate-reducing bacteria, no genes for porins have been identified or proposed in its annotated genome. Results from initial biochemical studies suggested that the product of the DVU0799 gene, which is one of the most abundant proteins of the D. vulgaris Hildenborough OM and purified as a homotrimeric complex, was a strong porin candidate. To investigate this possibility, this protein was further characterized biochemically and biophysically. Structural analyses via electron microscopy of negatively stained protein identified trimeric particles with stain-filled depressions and structural modeling suggested a β-barrel structure for the monomer, motifs common among the known porins. Functional studies were performed in which crude OM preparations or purified DVU0799 was reconstituted into proteoliposomes and the proteoliposomes were examined for permeability against a series of test solutes. The results obtained establish DVU0799 to be a pore-forming protein with permeability properties similar to those observed for classical bacterial porins, such as those of Escherichia coli. Taken together, these findings identify this highly abundant OM protein to be the major porin of D. vulgaris Hildenborough. Classification of DVU0799 in this model organism expands the database of functionally characterized porins and may also extend the range over which sequence analysis strategies can be used to identify porins in other bacterial genomes.

**IMPORTANCE** Porins are membrane proteins that form transmembrane pores for the passive transport of small molecules across the outer membranes of Gram-negative bacteria. The present study identified and characterized the major porin of the model sulfate-reducing bacterium Desulfovibrio vulgaris Hildenborough, observing its preference for anionic sugars over neutral ones. Its predicted architecture appears to be novel for a classical porin, as its core β-barrel structure is of a type typically found in solute-specific channels. Broader use of the methods employed here, such as assays for channel permeability and electron microscopy of purified samples, is expected to help expand the database of confirmed porin sequences and improve the range over which sequence analysis-based strategies can be used to identify porins in other Gram-negative bacteria. Functional characterization of these critical gatekeeping proteins from divergent Desulfovibrio species should offer an improved understanding of the physiological features that determine their habitat range and supporting activities.

## INTRODUCTION

Sulfate-reducing microorganisms are significant contributors to the mineralization of organic material in marine and freshwater sediments, as well as the terrestrial subsurface, oxidizing low-molecular-weight organic compounds via the respiratory reduction of sulfate to sulfide. Apart from serving a central role in biogeochemical transformations of carbon and sulfur, they are also known for their positive and negative impacts on natural and engineered systems. Their capacity to alter the toxicity and mobility of metals by complexation with sulfide or, in some cases, by direct reduction has been applied to environmental remediation and to the removal of metals from waste streams. The negative consequences of their activities include the souring of oil wells and the associated corrosion of oil recovery and delivery systems. These attributes have made the study of sulfate-reducing microorganisms a focus of both basic and applied research. Among sulfate-reducing microorganisms in culture, Desulfovibrio vulgaris Hildenborough has been one of the more extensively studied. This and other species of Gram-negative bacteria of the genus Desulfovibrio are widely distributed in the environment, and D. vulgaris Hildenborough has long served as a model for developing a better understanding of the biotic and abiotic factors that determine the distribution and activity of sulfate reducers (1–4).

An essential feature of Desulfovibrio and other Gram-negative bacteria is the presence of an outer membrane (OM), adding a second filter, in addition to the cytoplasmic membrane, for managing the passage of solutes into and out of the cell. Solute movement across the OM is facilitated by transmembrane channels, among which the porins form one of the most important families of bacterial membrane proteins (5, 6). These transmembrane channel-forming structures are homomeric complexes of β-barrel subunits each housing a water-filled pore that mediates the selective diffusion of a wide range of molecules across the OM. The amino acids lining these pores help define the steric and chemical constraints on permeability, passively regulating the flow of solutes, including nutrients and metabolites, while presenting an important obstacle to the entry of antibiotics. These channels of the OM have also been found to serve as docking sites or receptors for bacteriophages, like OmpF (a classical nonspecific, but cation-selective porin) does for bacteriophage K20 (7) and LamB (a maltodextrin specific channel) does for λ phage (8). Thus, these channels serve a central role in governing the interaction of Desulfovibrio bacteria with their environment.

Although of well-recognized physiological and environmental significance, no porins have been identified or proposed in the annotated genome of D. vulgaris Hildenborough available through major bioinformatics resources, such as UniProt (9, 10) or MicrobesOnline (11). Earlier biochemical analyses suggested that the product of the DVU0799 gene, one of the most abundant proteins of the D. vulgaris Hildenborough OM, was a porin candidate, existing as a homomeric trimer with monomers of about 51 kDa (12). To investigate this possibility, we further analyzed this protein using electron microscopy, amino acid sequence analysis, and molecular model-building methods. For the characterization of transmembrane channel functional properties, purified DVU0799 as well as the crude D. vulgaris Hildenborough OM were reconstituted into liposomes and evaluated for permeability using the liposome swelling assay (13).

## RESULTS

### Purification and biochemical characterization of DVU0799 identify a homotrimeric complex.

**FIG 1:**
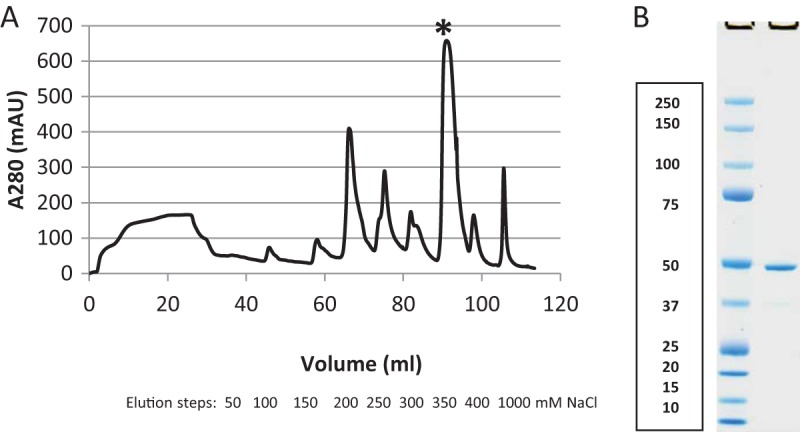
Isolation of DVU0799. (A) Anion-exchange chromatography elution profile. Samples were eluted from the column in 50 mM NaCl increments up to 400 mM, with the final elution step being with 1,000 mM NaCl. DVU0799 eluted primarily in the peak at 350 mM NaCl (at 90 ml [*]). mAU, milli-absorbance units. (B) SDS-PAGE of elution fraction sample. First lane, molecular mass standards (the numbers on the left are molecular masses [in kilodaltons]); second lane, sample of the fraction from the 350 mM NaCl elution peak.

The gene for DVU0799 yields a protein 466 amino acids in length containing an N-terminal signal sequence (residues 1 to 23) for initiating protein transport across the inner membrane (IM) (14), while the C-terminal region (residues 458 to 466) contains a signal sequence proposed to promote association with the β-barrel assembly machinery (BAM) complex and facilitate protein folding and assembly into the OM (15). DVU0799 is a highly abundant membrane protein of the D. vulgaris Hildenborough OM (12, 16) and can be isolated in significant quantities and a relatively pure form from wild-type cultures under standard growth conditions. For the studies described here, 0.3 to 0.5 g of cell membranes was processed per experiment. This amount of membrane could be obtained from the cells present in 3 to 5 liters of D. vulgaris Hildenborough wild-type cultures grown to mid-log phase. To begin the isolation process, washed cell membranes were pretreated with a relatively mild detergent, polyoxyethylene (9) dodecyl ether (C_12_E_9_) or sodium lauroyl sarcosinate (Sarkosyl), to solubilize and remove the proteins of the IM. Following ultracentrifugation, a portion of the remaining OM-enriched pellet (∼0.1 g) was set aside for liposome incorporation, with the remainder being solubilized for protein purification using the detergent octyl glucoside (OG). Ion-exchange chromatography of the solubilized OM components yielded fractions enriched with DVU0799 eluting at the 350 mM NaCl step (Fig. 1A). Sodium dodecyl sulfate (SDS)-polyacrylamide gel electrophoresis (PAGE) of fractions around this elution peak showed that they contained relatively pure DVU0799 (mass spectrometry confirmation; data not shown) with an apparent molecular mass of about 50 kDa (Fig. 1B).

**FIG 2:**
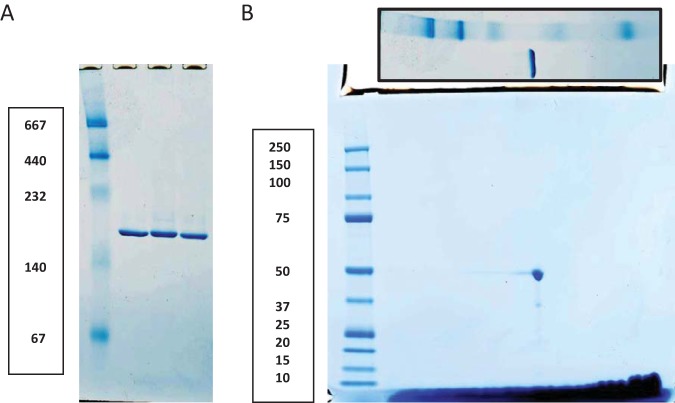
Blue native PAGE and second-dimension SDS-PAGE. (A) BN-PAGE of DVU0799 samples (second to fourth lanes). The first lane contains the molecular mass standards. (B) 2D SDS-PAGE of the native form of DVU0799. As a guide for the horizontal molecular mass distribution, an image of the corresponding native gel lane with mass markers was placed along the top of the 2D gel. Taken together, these gels are consistent with DVU0799 forming a homotrimeric complex in the native state. In both panels, the numbers on the left are molecular masses (in kilodaltons).

To examine the native state characteristics of this protein, we subjected samples of the ion-exchange elution fractions containing DVU0799 to blue native PAGE (BN-PAGE). Operating under conditions found to support the preservation of membrane protein oligomeric states (in a cold room overnight at 70 V [12]), the BN-PAGE samples ran at a molecular mass of about 170 kDa (Fig. 2A). To assess the composition of the complex, lanes cut from these native gels were further processed using a second-dimension (2D) SDS-PAGE step (Fig. 2B). The 2D SDS-PAGE results indicated that the protein band seen on the BN-polyacrylamide gel is a homomeric complex of DVU0799. Taking into account the potential mass contributions from Coomassie blue dye micelles (10 to 20 kDa), the apparent native molecular mass suggests that DVU0799 forms a homotrimeric complex, a quaternary structure motif common among bacterial porins.

### Structural modeling suggests an 18-strand β-barrel motif for the DVU0799 monomer.

**FIG 3:**
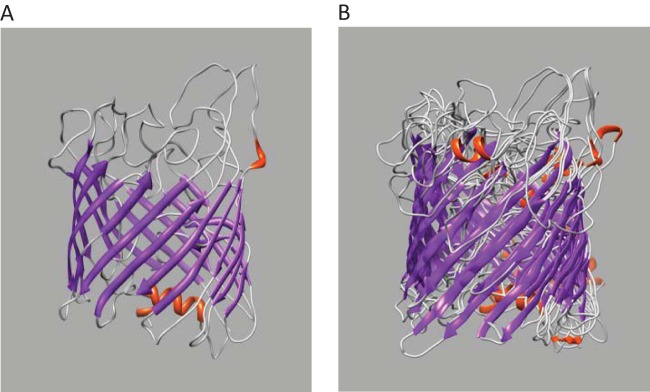
I-TASSER-based structural models of DVU0799. (A) Top solution; (B) aligned family of solutions. The core structure is that of an 18-strand β-barrel. This structural motif is similar to that found in the subunits of previously characterized bacterial porins. The structure is depicted in ribbon format and colored to identify regions of secondary structure: gray, random coil; purple, β-strand; red, α-helical regions.

To determine if structural modeling of the DVU0799 sequence could provide additional insight into the general structure and potential function of this OM protein, the amino acid sequence in FASTA format was processed through the I-TASSER protein modeling server (https://zhanglab.ccmb.med.umich.edu/I-TASSER) (17–19). The I-TASSER modeling output included four candidate structures (Protein Data Bank [PDB] format) with C scores ranging from −2.41 to −1.67. The C scores assigned by the program ranged from −5 to 2, where a C score of a higher value indicates a model of higher confidence. As C-score values of −1.5 and higher are generally indicative of the correct fold (18), a score of −1.67 for the top DVU0799 model, particularly given the high percentage of predicted coils (∼50%), is reflective of a reliably predicted β-barrel core structure (Fig. 3A). These models were visualized and figures prepared using the UCSF Chimera molecular modeling program (http://www.cgl.ucsf.edu/chimera) (20).

Comparison of these predicted structures found them to be similar overall and to feature an 18-strand β-barrel core structure, varying predominately in the shape and length of the random coil and connecting loop regions at the extramembrane faces and within the cylinder. Figure 3A shows the structure of the top-ranked result, while Fig. 3B shows the family of proposed solutions superimposed and aligned. The models are depicted in ribbon format, with the secondary structure features being color coded (gray, random coil; purple, β-strand; red, α-helix). While the accuracy of the location and orientation of specific amino acids, particularly those of the predicted random coil regions, remains to be determined, the consistent identification of a β-barrel core as the central architectural motif is significant. This feature is the classic structural element of the bacterial porin monomer structures previously determined by X-ray crystallographic methods (21–23).

The database entry for DVU0799 in the UniProt Knowledgebase (UniProtKB; www.uniprot.org [9, 10]) describes it as an “uncharacterized protein,” and in MicrobesOnline (www.microbesonline.org [11]) it is designated a “conserved hypothetical protein.” A BLAST-based search (24, 25) comparing the DVU0799 amino acid sequence against the amino acid sequences of all protein entries in UniProtKB identified almost 60 proteins having a sequence identity with DVU0799 of 40% or greater. This group is predominantly populated with proteins annotated as “uncharacterized.” However, five entries (UniProt accession numbers G2HC51, G2HB34, G2HC52, B2KPH3, and B7SKM7) are annotated as porins. Of these, only the assignments of the sequences with accession numbers B2KPH3 (Bilophila wadsworthia) and B7SKM7 (Desulfovibrio piger), each sharing about 43% sequence identity with DVU0799, are based on direct experimental studies (26).

### Electron microscopy of DVU0799 identifies trimers of stain-filled depressions.

**FIG 4:**
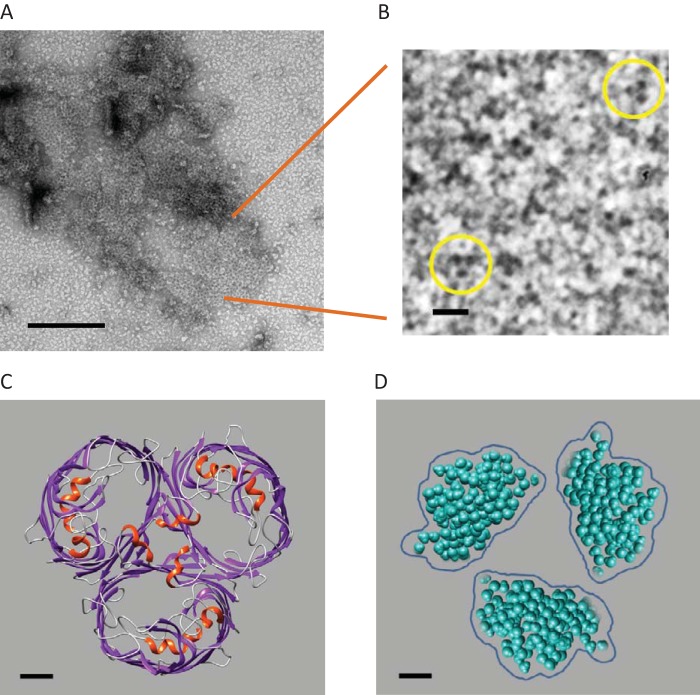
Electron microscopy of DVU0799-containing fractions. (A) Wide-area view of a negatively stained preparation enriched with DVU0799 protein associating to form honeycomb-like arrays. Bar, 200 nm. (B) Enlarged view of the honeycomb-like region in which trimers of stain-filled depressions are highlighted (circles). Bar, 10 nm. (C) A typical example of a classical (nonspecific) porin, OmpF of E. coli (PDB accession number 1OPF), is shown in top view, perpendicular to the membrane plane. The trimer structure is based on a homomeric complex of three β-barrels, each forming a transmembrane channel. The structure is depicted in ribbon format and colored to identify regions of secondary structure: gray, random coil; purple, β-strand; red, α-helical regions. Bar, 1 nm. (D) The waters able to occupy the solvent-accessible regions of the pores, indicative of where stain molecules would be expected to accumulate, are shown to aid comparison of the porin model in panel C with the trimers of stain-filled pores seen in panel B. The general size and shape of the water-filled pores in this view are similar to the stain-filled regions observed in the images of negatively stained DVU0799 trimers. Waters were placed within the channels using the Solvate utility of the Chimera program. Bar, 1 nm.

Samples of ion-exchange chromatography fractions enriched with DVU0799 were negatively stained (2% uranyl acetate) and examined by transmission electron microscopy. Large patches or pseudoarrays of proteins associating to form honeycomb-like lattices were found throughout the sample preparations (Fig. 4A). Upon closer examination of these regions, trimers of stain-filled depressions could be seen (Fig. 4B; highlighted by yellow circles). These trimers have an effective diameter of about 8 nm and a 3.5 nm center-to-center distance between stain-filled depressions. For comparison, a structural model of E. coli OmpF as a typical example of a classical porin (PDB accession number 1OPF) is shown in top view (Fig. 4C) (21). Solvent-accessible regions of the pores in this structure are highlighted by separately displaying the waters able to occupy these spaces; the distribution of water is indicative of where stain molecules would be expected to accumulate (Fig. 4D). Collectively, the images of negatively stained samples and the general structures of OmpF and its pore waters show that the trimers of DVU0799 have a size and geometry similar to those of previously characterized classical porins (27, 28).

### Permeability of proteoliposomes prepared with crude D. vulgaris Hildenborough OM or purified DVU0799.

To assess whether DVU0799 exhibits membrane channel properties characteristic of bacterial porins, we utilized the liposome swelling assay (13) to examine the solute permeability of proteoliposomes into which D. vulgaris Hildenborough OM proteins were reconstituted, using either crude OM preparations or purified protein. In this approach, the degree of membrane channel permeability is reflected in the rate of proteoliposome swelling occurring in response to the influx of water coupled with the movement of test solutes across the proteoliposome membranes. Swelling rates were experimentally determined by monitoring the decrease in the optical density (OD), measured at a source wavelength of 400 nm (OD_400_), of the proteoliposome and test solute mixtures over time. For plotting and comparisons of relative permeability, swelling rates were scaled by adjusting the value obtained for the relatively small uncharged sugar l-arabinose, at the upper limit of measureable permeability, to a value of 100 and scaling the values for the other solutes accordingly.

**FIG 5:**
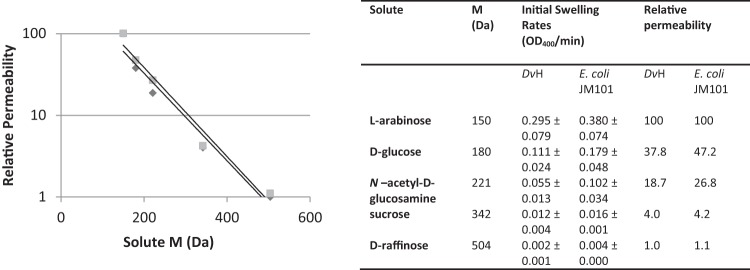
D. vulgaris Hildenborough (*Dv*H) and E. coli relative OM permeability. Initial liposome swelling rates were measured using proteoliposomes reconstituted with whole OM from D. vulgaris Hildenborough or E. coli (experimental averages and standard deviations are listed). The solutes evaluated were l-arabinose (150 Da), d-glucose (180 Da), *N*-acetyl-d-glucosamine (221 Da), sucrose (342 Da), and d-raffinose (504 Da). Relative permeability values were derived from the swelling rate averages, scaled so that l-arabinose was assigned a value of 100. Trend lines for both sets of relative permeabilities are shown. Results indicate that for the conditions surveyed the overall OM permeabilities for the two microbes are similar. Diamonds, D. vulgaris Hildenborough; squares, E. coli JM101. M, molecular mass.

As the native complex of DVU0799 was found to be more detergent sensitive than previously characterized porins, a set of preliminary experiments in which permeability could be evaluated using proteoliposomes prepared with crude D. vulgaris Hildenborough OM preparations was conducted. Here, whole-cell membranes were subjected to only a relatively mild detergent step for the removal of IM components. In this way, OM proteins could remain within a lipid bilayer environment throughout the process, improving the likelihood that these proteins would retain their structure and function. This approach allowed us to assess the general suitability of the liposome swelling method for D. vulgaris Hildenborough OM protein characterization and permitted comparisons of the permeability of D. vulgaris Hildenborough crude OM to that of E. coli crude OM-based proteoliposomes prepared in a similar manner (Fig. 5). The relative permeabilities for proteoliposomes exposed to a series of uncharged sugars of increasing molecular mass (l-arabinose, 150 Da; d-glucose, 180 Da; *N*-acetyl-d-glucosamine, 221 Da; sucrose, 342 Da; d-raffinose, 504 Da) were determined.

From these experiments it is evident that the general permeability profile of the D. vulgaris Hildenborough OM, at least with respect to this solute test set, is comparable to that of the E. coli OM. Of the candidate porin gene products in the D. vulgaris Hildenborough OM (DVU0799, DVU0797, DVU0371, DVU0273), DVU0799 is by far the most abundant (12, 16). Its high-level presence suggests that the overall permeability of the D. vulgaris Hildenborough OM is dominated by the performance of DVU0799.

Given that the assay was effective in assessing D. vulgaris Hildenborough crude OM permeability, the investigation was extended to studies of proteoliposomes reconstituted with purified DVU0799. In early experiments on porin permeability, such as those first characterizing the properties of E. coli, Salmonella enterica serovar Typhimurium, and Pseudomonas aeruginosa proteins, porins were often extracted for liposome reconstitution using sodium dodecyl sulfate (SDS) as the solubilizing detergent (13, 29, 30). The DVU0799 trimeric complex, however, is not similarly stable in SDS and requires a milder detergent to facilitate intact complex solubilization and purification. Therefore, crude D. vulgaris Hildenborough OM preparations like those used in the initial experiments described here were solubilized with the detergent OG, noted for its effectiveness in the stable solubilization of OM proteins (26, 31, 32). The properties of OG, such as its higher critical micelle concentration, allowed the more complete removal of detergent upon reconstitution into proteoliposomes but also required care to avoid removing detergent too rapidly and precipitating protein from solution.

**FIG 6:**
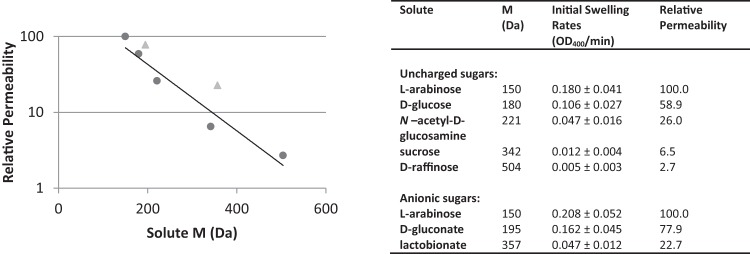
Permeability of proteoliposomes reconstituted with purified DVU0799. Initial swelling rates obtained with five uncharged sugars of increasing molecular mass (l-arabinose, 150 Da; d-glucose, 180 Da; *N*-acetyl-d-glucosamine, 221 Da; sucrose, 342 Da; d-raffinose, 504 Da) and two anionic sugars (d-gluconate, 195 Da; lactobionate, 357 Da) were measured using proteoliposomes reconstituted with purified DVU0799; experimental averages and standard deviations are listed. Relative permeability is scaled to that for l-arabinose, assigned a value of 100. The trend line for the relative permeability values for uncharged sugars is shown. Results suggest a modest preference for the transport of anionic sugars over uncharged sugars of similar mass. Circles, uncharged sugars; triangles, anionic sugars.

Purified DVU0799 solubilized in OG was successfully reconstituted into proteoliposomes and studied for solute uptake and swelling using the same five uncharged sugars tested against the proteoliposomes prepared with the D. vulgaris Hildenborough crude OM. The results obtained demonstrated that DVU0799 provided the proteoliposomes with the permeability characteristics typically observed for bacterial porins (Fig. 6). As can be seen on the left of Fig. 6, the permeability profile begins with that for l-arabinose at the upper limit of the swelling rate measurement, assigned a relative permeability value of 100, and this value drops almost 2 orders of magnitude to approach the permeability for the trisaccharide d-raffinose.

This profile indicates permeability rates for this set of proteoliposomes and test solutes moderately higher than those that were observed for proteoliposomes reconstituted from crude OM preparations (Fig. 5). This may in part be because the concentration of DVU0799 present in crude OM preparations is lower than that present in the purified sample, resulting in larger amounts of DVU0799 reconstituted into proteoliposomes prepared from purified protein. An impact on porin functional properties due to isolation from the native bilayer lipid environment could also be a contributing factor.

Proteoliposomes reconstituted with purified DVU0799 were also surveyed for their permeability to two sugar acids, d-gluconate (195 Da) and lactobionate (357 Da), whose molecular masses fall within the molecular mass range covered by the uncharged sugars examined (Fig. 6). In a comparison of relative permeabilities, the results suggest a modest preference for the transport of anionic sugars over uncharged sugars of similar mass.

## DISCUSSION

D. vulgaris Hildenborough has been a model organism for the study of sulfate-reducing bacteria and investigations of its role in corrosion and potential for use in bioremediation applications (1–4), yet in spite of the extensive study of this bacterium, no gene encoding a porin had been identified in its genome sequence. Results from an earlier study of D. vulgaris Hildenborough OM protein complexes identified several candidate porin genes (12). The protein product of the DVU0799 gene was found to be the most abundant of these, and perhaps more importantly, it is one of the most abundant proteins of the D. vulgaris Hildenborough OM overall. Long-standing efforts to obtain DVU0799 transposon-based (33) or deletion mutant strains have been unsuccessful, suggesting that the gene product is essential.

In the present study, we investigated DVU0799 biochemically and biophysically, examining purified samples via blue native PAGE and 2D SDS-PAGE, imaging samples by the use of negative-stain electron microscopy and through sequence analysis and structural modeling. Structural modeling yielded predictions of an 18-strand β-barrel as the core motif, while electron microscopy of preparations enriched with DVU0799 homotrimeric complexes revealed trimers of stain-filled depressions approximately 8 nm in diameter, classic hallmarks of bacterial porins. We also examined DVU0799 for transmembrane channel function by reconstituting purified protein into proteoliposomes and monitoring swelling rates following their introduction into solutions containing test solutes. It was initially unclear whether this method would be effective in evaluating the permeability of detergent-solubilized DVU0799, which, in contrast to porins previously studied, was not similarly stable in SDS. To examine whether D. vulgaris Hildenborough porin complexes retained their function following the initial step of mild detergent treatment (using Sarkosyl or C_12_E_9_) for removal of IM components, the resulting crude OM preparations (where OM proteins are maintained within lipid bilayers) were reconstituted into proteoliposomes and evaluated. The results obtained showed that the transmembrane channels of the OM remained functional. The permeability of the crude D. vulgaris Hildenborough OM was found to be similar to that of crude E. coli OM over the range of solutes tested. Next, DVU0799 was extracted from its native lipid bilayer environment in the crude OM using a relatively mild nonionic detergent, OG, and purified. The trimeric structure of DVU0799 was maintained using OG, as evidenced by blue native PAGE and electron microscopy results, and the complex was functionally preserved so that when it was reconstituted, the protein displayed the permeability characteristics expected for a porin-type transmembrane channel. Notably, sugar acids were found to permeate the channel more rapidly than uncharged sugars of similar size. In the case of D. vulgaris Hildenborough, having a high nutrient demand for sulfate, this preference for anions may be beneficial for growth under conditions where sulfate availability is low. Given the aforementioned suggestion that the DVU0799 gene is essential, DVU0799 may play a critical role in the uptake not only of sulfate but also of other solutes. The structural and functional results presented here establish DVU0799 as a pore-forming protein with size, shape, and permeability properties similar to those observed for classical bacterial porins. Given the relatively high abundance of this protein among all D. vulgaris Hildenborough OM proteins, these findings identify this protein as the major porin of this sulfate-reducing bacterium.

In a pioneering study, Avidan and colleagues identified and characterized the first examples of porins from the family Desulfovibrionaceae (26). Their work targeted the structure and function of candidate porins, Omp-DP and Omp-BW, from Desulfovibrio piger and Bilophila wadsworthia, respectively. While the sequence homology of these porins to the DVU0799 amino acid sequence falls in the range of 40 to 45% identity, similarity to these and other known porins has not been sufficient for automated gene annotation approaches to identify DVU0799 as a porin. Furthermore, while the monomer structures for D. piger, B. wadsworthia, and D. vulgaris Hildenborough all appear to be based on 18-strand β-barrel motifs, there are substantial differences in their permeability. Although the DVU0799 pore is permeable to trisaccharides (i.e., d-raffinose, 504 Da), D. piger and B. wadsworthia were found to be impermeable to even disaccharides (sucrose, 342 Da; maltose, 360 Da), indicating a substantially smaller permeability limit and suggesting important differences in pore-defining structures. It should be noted that although all of these porins are from members of the family Desulfovibrionaceae, these proteins also come from bacteria cultivated from two widely different environments: D. vulgaris Hildenborough was isolated from soil (34), while D. piger and B. wadsworthia were isolated from human patients (35, 36).

As a means for exploring structure-function relationships in this set of porins from the class Deltaproteobacteria, the recently determined structure of the major outer membrane protein (MOMP) porin from the epsilonproteobacterium Campylobacter jejuni (37), which features a number of similarities with D. vulgaris Hildenborough porin DVU0799, is an informative example. As was found to be the case with DVU0799 and D. vulgaris Hildenborough, the MOMP porin is the major porin of C. jejuni, it is also a homotrimer in which its monomers are based on a β-barrel core composed of 18 strands, and it is anion selective. The 18-strand β-barrel motifs found for the MOMP porin and predicted for DVU0799 are noteworthy, for these porins display permeability properties similar to those observed for the classical or general porins, which exclusively feature 16-strand β-barrels; core β-barrel structures based on 18-strand motifs have, prior to the determination of the structure of the MOMP porin, been noted only in solute-specific channels. From a structural stability standpoint, echoing our experience with DVU0799, the trimer structure of MOMP is sensitive to even modest SDS treatment, particularly in comparison to the general porins, which can require heating of samples to greater than 70°C even in the presence of 2% SDS to completely dissociate the trimers (38, 39).

Taking into consideration the potential for overinterpretation of a predicted model structure, even one with a relatively good confidence score, it remains worthwhile to note a further similarity with the C. jejuni porin. MOMP and the predicted structure for DVU0799 share, along with the general porins, an overall pattern of charged residue distribution around the permeability-defining constriction region, or eyelet, of their respective channels. The negatively charged residues located on a pore's internalized loop regions (commonly, loop L3, but also loop L4 in the case of MOMP) establish a net negative charge on one side of the eyelet, while across the pore from this region, the inner β-barrel wall is lined with a predominance of positively charged residues. Through this separation of charge, a transverse electric field helping to shape channel selectivity is established across the eyelet. What sets MOMP apart, however, is an arrangement of negatively charged side chains and carbonyl groups on the loop segments of the constriction region that is able to bind a Ca^2+^ ion with a high affinity. With Ca^2+^ bound, channel permeability changes from what would be moderately cation selective to anion selective. Given the aforementioned similarities between MOMP and DVU0799, it is interesting to consider the possibility that some form of constriction region cation binding site also plays a role in establishing the preference for anionic sugars observed for DVU0799.

High-resolution structural data remain an important tool for interpreting potential gene product function, especially when the homology of the amino acid sequence to the sequences of functionally related structures is low. However, the collection of such data continues to be challenging. By compiling sets of often more readily obtainable functional and lower-resolution structural data (e.g., by negative-stain electron microscopy), gene descriptions can still be developed and assigned with confidence. It is hoped that broader use of the approach employed here and in the work of Avidan et al. (26) for the identification of porins from members of the Desulfovibrionaceae family will continue to expand the database of confirmed porin sequences and signatures and increase the range over which sequence analysis-based strategies can be used to identify porins in other Gram-negative bacteria. On a more specific level, the functional characterization of these critical gatekeeping proteins from divergent Desulfovibrio species should offer an improved understanding of the physiological features that determine their habitat range and supporting activities.

## MATERIALS AND METHODS

### Cell growth.

Working within an anaerobic chamber (Coy Lab Products), D. vulgaris Hildenborough (ATCC 29579) batch cultures were initiated from 15% glycerol freezer stocks inoculated into Balch tubes (18- by 150-mm glass tubes with a narrow opening to hold a 1-in.-thick rubber septum) containing coculture medium A (which consists of, per liter, 2.3 g NaCl, 5.5 g MgCl_2_·6H_2_O, 0.14 g CaCl_2_·2H_2_O, 0.5 g NH_4_Cl, 0.1 g KCl, 4.32 ml 60% sodium dl-lactate, 1 mg resazurin, 0.192 g K_2_HPO_4_, 2.1 g NaHCO_3_, 1 ml trace minerals, 1 ml Thauer's vitamins, 0.18 g l-cysteine hydrochloride, and 0.078 g NaS·9H_2_O, pH 7.2) (40) with 4.3 g/liter NaSO_4_ added and an 80% N_2_- and 20% CO_2_-filled headspace. The starter culture density was spectrophotometrically monitored (by determination of the OD_600_), and upon reaching the end of exponential growth, 3 ml of culture was removed and used to inoculate 3 liters of medium in two stoppered 2-liter bottles (each containing 1.5 liters of medium). These batch cultures were grown to stationary phase at 37°C and anoxically transferred into 500-ml sterile centrifuge bottles. The biomass was pelleted by centrifugation at 9,000 × *g* for 30 min at 4°C. Following centrifugation, the supernatant was removed and the cell pellet was resuspended in 50 ml of a basal salt solution. The resuspended cells were anoxically transferred to a 50-ml conical tube and subjected to centrifugation at 18,500 × *g* for 10 min at 4°C. Cell pellets were frozen in liquid nitrogen and stored at −80°C.

For the culturing of E. coli K-12 JM101 (New England BioLabs), colonies grown on LB agar plates were used to inoculate LB medium (50 ml in 250-ml flasks), following which the flasks were placed in a shaker for growth overnight at 30°C. On the following day, flasks containing 1.5 liters LB medium were inoculated with 15 ml of the overnight culture and again placed in a shaker at 30°C. Upon reaching an OD_600_ of ∼1.3, cells were harvested from the culture medium by low-speed centrifugation (8,000 × *g* for 15 min). Cell pellets were frozen in liquid nitrogen and stored at −80°C.

### Cell membrane isolation.

To reduce the level of iron sulfide present as a consequence of growth in modified coculture medium A, D. vulgaris Hildenborough cells were washed in cell wash buffer (20 mM HEPES, pH 7.4, 2 mM NaN_3_, 100 mM KCl, 0.1 mM EDTA, 1 mM MgCl_2_, 125 mM sucrose, 1 mM phenylmethylsulfonyl fluoride [PMSF]) prior to lysis. Cell pellets were resuspended in cell wash buffer (typically, 10 to 15 ml buffer per gram of pellet) and gently stirred at 4°C until a uniform suspension was obtained. A broad-spectrum protease inhibitor (cOmplete; Roche) was added to the wash and lysis buffers. To obtain a washed cell pellet, the cell suspension was centrifuged at 10,000 × *g* for 10 min at 4°C. Washed cell pellets were resuspended in lysis buffer (about 20 ml of 25 mM HEPES, pH 7.6, 100 mM KCl, 12.5 mM MgCl_2_, 0.1 mM EDTA, 20% glycerol) and processed through a gas-driven cell disruptor (EmulsiFlex-C5; Avestin) three times to break open the cells. To enhance the preservation of membrane protein complexes, the cell disruptor was chilled with ice. The broken cell suspension was centrifuged at low speed (10,000 × *g* for 10 min at 4°C) to remove unbroken cells; the supernatant from this step was centrifuged at high speed (100,000 × *g*, 1 h, 4°C) to isolate membranes. To reduce the presence of highly abundant soluble proteins, the membrane pellet was resuspended and washed several times in membrane wash buffer (20 mM HEPES, pH 7.4, 2 mM NaN_3_, 100 mM NaCl, 1 mM MgCl_2_, 0.1 mM EDTA, 1 mM PMSF). After each wash, the membranes were pelleted by high-speed centrifugation (100,000 × *g*, 1 h, 4°C). The membranes were either used immediately or quick-frozen and stored at −80°C. Cell membranes were prepared from E. coli K-12 JM101 cell pellets in a similar manner.

### Membrane processing.

To obtain cleaner preparations of outer membrane (OM) protein prior to chromatography, proteins were extracted from D. vulgaris Hildenborough cell membranes in a two-step process, generally based on the protocol of Baldermann et al. (41). In this sequential approach, solubilization of predominantly inner membrane (IM) proteins is performed in the initial step, followed by solubilization of the remaining, predominantly OM protein, in the second step. To extract proteins of the IM, washed membranes (typically, 0.3 to 0.5 g) were placed into a hand homogenizer along with about 10 ml of solubilization buffer (20 mM HEPES, pH 7.4, 2 mM NaN_3_, 100 mM NaCl, 1 mM MgCl_2_, 0.2 mg/ml lysozyme) containing 0.1% polyoxyethylene (9) dodecyl ether (C_12_E_9_) or 0.5% sodium lauroyl sarcosinate (Sarkosyl), and homogenized on ice for 1 h. In general, detergents and buffers were adjusted to obtain a final detergent-to-protein ratio of about 1:1 while maintaining a protein concentration of at least 10 mg/ml. After solubilization, the detergent concentration was lowered by dilution with solubilization buffer, and the sample was centrifuged (100,000 × *g* for 1.5 h at 4°C) to pellet unsolubilized membranes enriched in OM proteins. For our studies, proteoliposomes were prepared using either these enriched membranes directly or protein purified from these membranes. E. coli K-12 JM101 OM protein-enriched membranes were prepared in a similar fashion with the use of 0.5% Sarkosyl. To extract proteins of the D. vulgaris Hildenborough OM, the resulting D. vulgaris Hildenborough membrane pellets were processed in solubilization buffer (about 10 ml) containing a more active detergent, octyl glucoside (OG; up to 3.4%), and homogenized on ice for 1 h. After solubilization, the detergent concentration was lowered by dilution with solubilization buffer and the sample was centrifuged (100,000 × *g* for 1.5 h at 4°C). Proteins in the supernatant were then subjected to ion-exchange chromatography.

### Ion-exchange chromatography purification of DVU0799.

For purification of OM proteins from 0.3 to 0.5 g of treated membranes, a 2-ml bed of anion-exchange medium (Q Sepharose HP; GE Healthcare) was equilibrated in buffer A (20 mM HEPES, pH 7.4, 2 mM NaN_3_) containing 1% OG. Detergent-solubilized samples were loaded onto the column, and the column was washed to remove nonspecifically bound material using buffer A. Bound proteins were eluted in 8- to 12-ml steps at 50 mM NaCl increments over a range of from 50 to 400 mM NaCl, followed by a final step of elution with 1 M NaCl. The elution buffer was prepared by mixing buffer A with increasing levels of buffer B (buffer A with 1 M NaCl added).

### Blue native PAGE and SDS-PAGE.

Blue native polyacrylamide gel electrophoresis (PAGE) of the ion-exchange chromatography fractions was performed on the basis of the protocol of Schägger et al. (42) with modifications. Briefly, 45-μl OM preparation samples were mixed with 5 μl of glycerol and 3 to 4 μl of a stock of 0.5% Coomassie G-250 in 1 M aminocaproic acid. Precast acrylamide gels (4 to 12% bis-Tris gels, 1.0 mm; Invitrogen) were equilibrated in a cathode buffer (50 mM Tricine, 15 mM bis-Tris, pH 7.0) containing 0.05% dodecyl maltoside (DDM) and an anode buffer of 50 mM bis-Tris, pH 7.0. When the gel was run with the samples loaded, the cathode buffer was replaced with a solution containing 50 mM Tricine, 15 mM bis-Tris, 0.02% Coomassie G-250, and 0.05% DDM, pH 7.0. The gels were run overnight at 70 V and 4°C. Whole lanes 6 cm in length and 0.5 cm in width were cut from the native gels and placed length-wise across the tops of gels containing one wide sample well in addition to the molecular mass standard well (4 to 12% bis-Tris; 1.5 mm; 2D; Invitrogen) for SDS-PAGE. Native gel lane strips were incubated in Laemmli sample buffer (Sigma) for 20 min prior to running the SDS-polyacrylamide gels with a MOPS (morpholinepropanesulfonic acid)-based buffer (MOPS-SDS running buffer; Invitrogen) according to the manufacturer's directions.

### Electron microscopy.

Specimens (4 μl) were placed on glow-discharged carbon-coated electron microscopy grids (Formvar-carbon, 200 mesh; Electron Microscopy Sciences). After 5 min, the sample on the grid was blotted to less than 1 μl and the grid was placed on an ultrapure water droplet, sample side down, to reduce the level of salts on the grid. The water droplets used were 50 to 100 μl in volume and placed on a sheet of Parafilm. The grid was subsequently transferred to two more water droplets following wait periods of 2 min each. After the last water wash step, the grid was removed from the droplet and inverted. The water drop remaining on the grid was blotted to below 1 μl, after which 2 μl of 2% uranyl acetate was added. After 1 min the grid was blotted to dryness. Sample grids were examined in a JEOL JEM-1200x electron microscope operating at 100 kV. Images were recorded using a 2,000- by 2,000-pixel charge-coupled-device camera (UltraScan; Gatan) controlled by the DigitalMicrograph software package.

### Structural modeling and sequence analysis.

A FASTA format amino acid sequence of DVU0799 was submitted to the I-TASSER server (https://zhanglab.ccmb.med.umich.edu/I-TASSER) (17–19) for modeling using the default parameters. PDB files generated for the predicted structures were visualized via the UCSF Chimera program (http://www.cgl.ucsf.edu/chimera) (20). Waters were placed within the channels using the Solvate utility of the Chimera program. The DVU0799 amino acid sequence was also sent for BLAST analysis (24, 25) and comparison against the amino acid sequences of all UniProt protein entries (9, 10) via the UniProtKB webpage for DVU0799 using the default run parameters.

### Liposome swelling assay.

The procedures used for the liposome swelling assay were based on earlier work by Nikaido and Rosenberg (13). In this approach, porin permeability is regarded to be proportional to the rate of liposome (into which porins have been reconstituted) swelling and the swelling rate is proportional to the change in optical density over time. The rates determined are scaled to form a set of relative permeabilities adjusted so that the rate for the sugar arabinose is fixed at 100. For initial studies of uncharged solute transport, proteoliposomes were made by combining 2.4 μmol of egg phosphatidylcholine (Avanti) and 0.15 μmol dicetyl phosphate (Sigma) in 0.3 ml of 15% (wt/vol) Dextran T-40 (Sigma) in 10 mM Tris-HCl buffer at pH 8.0 with crude D. vulgaris Hildenborough OM (2 to 5 μg total protein), crude E. coli K-12 JM101 OM (0.5 to 2.5 μg total protein), or purified DVU0799 (0.5 to 1 μg protein). Control liposomes were prepared without the addition of protein. Portions (17 μl) of a liposome suspension were added to a cuvette containing 0.6 ml of a solution of about 20 mM uncharged sugar test solute in 10 mM Tris-HCl buffer, pH 8.0. The optical density at 400 nm was continuously recorded with a spectrophotometer (UVIKON860; Kontron Instruments). Test solute concentrations were adjusted as needed to ensure that the external solution was isotonic with the control liposomes. For experiments evaluating porin permeability for anionic as well as uncharged solutes, liposomes were made by combining 6.2 μmol of egg phosphatidylcholine and 0.2 μmol of dicetyl phosphate in 0.4 ml of a solution containing 12 mM stachyose (Sigma), 4 mM sodium NAD (Sigma), and 1 mM imidazole-NAD (Sigma), pH 6.0. For the preparation of proteoliposomes in this series of experiments, 10 to 25 μg of purified DVU0799 was included. For each measurement, 17 μl of the proteoliposome suspension was added to a cuvette containing either 0.6 ml of an approximately 20 mM solution of an uncharged sugar or a 9 mM solution of a monovalent anionic sugar in 4 mM sodium NAD (Sigma) and 1 mM imidazole-NAD (Sigma), pH 6.0; the optical density was recorded as described above. The uncharged sugars evaluated were l-arabinose (Sigma), d-glucose (Sigma), *N*-acetyl-d-glucosamine (Sigma), sucrose (Sigma), and d-raffinose (Sigma). The anionic sugars tested were sodium salts of d-gluconate (Sigma) and lactobionate (Glentham Life Sciences).
